# Modulation of cell cycle increases CRISPR-mediated homology-directed DNA repair

**DOI:** 10.1186/s13578-023-01159-4

**Published:** 2023-11-25

**Authors:** Guoling Li, Xiaohui Yang, Xinxin Luo, Zhenfang Wu, Huaqiang Yang

**Affiliations:** 1 National Engineering Research Center for Breeding Swine Industry, College of Animal Science, South China Agricultural University, Guangzhou, China; 2 Yunfu Branch Center of Guangdong Laboratory of Lingnan Modern Agricultural Science and Technology, Yunfu, 527400 China; 3 Present Address: Institute of Neuroscience, State Key Laboratory of Neuroscience, Key Laboratory of Primate Neurobiology, CAS Center for Excellence in Brain Science and Intelligence Technology, Shanghai Institutes for Biological Sciences, Chinese Academy of Sciences, Shanghai, China

**Keywords:** Gene editing, Homology-directed repair, Cell cycle, CRISPR/Cas9, CDK1, CCNB1

## Abstract

**Background:**

Gene knock‐in (KI) in animal cells via homology‐directed repair (HDR) is an inefficient process, requiring a laborious work for screening from few modified cells. HDR tends to occur in the S and G2/M phases of cell cycle; therefore, strategies that enhance the proportion of cells in these specific phases could improve HDR efficiency.

**Results:**

We used various types of cell cycle inhibitors to synchronize the cell cycle in S and G2/M phases in order to investigate their effect on regulating CRISPR/Cas9-mediated HDR. Our results indicated that the four small molecules—docetaxel, irinotecan, nocodazole and mitomycin C—promoted CRISPR/Cas9-mediated KI with different homologous donor types in various animal cells. Moreover, the small molecule inhibitors enhanced KI in animal embryos. Molecular analysis identified common signal pathways activated during crosstalk between cell cycle and DNA repair. Synchronization of the cell cycle in the S and G2/M phases results in CDK1/CCNB1 protein accumulation, which can initiate the HDR process by activating HDR factors to facilitate effective end resection of CRISPR-cleaved double-strand breaks. We have demonstrated that augmenting protein levels of factors associated with the cell cycle via overexpression can facilitate KI in animal cells, consistent with the effect of small molecules.

**Conclusion:**

Small molecules that induce cell cycle synchronization in S and G2/M phases promote CRISPR/Cas9-mediated HDR efficiency in animal cells and embryos. Our research reveals the common molecular mechanisms that bridge cell cycle progression and HDR activity, which will inform further work to use HDR as an effective tool for preparing genetically modified animals or for gene therapy.

**Supplementary Information:**

The online version contains supplementary material available at 10.1186/s13578-023-01159-4.

## Introduction

Gene knock-in (KI) manipulation can precisely confer predefined modifications to genome, and thus is valuable for establishment of genetically engineered cells or animals with desired genotypes and gene therapy of certain genetic diseases. The fact is that KI occurs in a low efficiency in animal cells [1]. Occurrence of KI relies on creation of target double-strand breaks (DSBs) and homology-directed repair (HDR) pathway to mend DSB, both of which are quite inefficient. Use of site-specific nucleases, such as CRISPR/Cas9, can induce target DSB with a high frequency, therefore promoting the following DSB repair efficiency [2]. However, HDR remains difficult to be improved considerably. HDR requires the presence of homologous template for a precise repair. In animal cells, HDR is competed by non-homologous end joining (NHEJ) pathway, a predominant but error-prone DSB repair process in animal cells. In addition, NHEJ can occur throughout the entire cell cycle, whereas HDR mainly occurs in late S and G2/M phases of cell cycle [3–5]. These elements collectively limited HDR frequency in animal cells.

A previous work showed that a small molecule, nocodazole (NOC), which induces cell cycle arrest at G2/M phase, can significantly enhance HDR in multiple animal cell types [6], presenting a simple and effective strategy to facilitate precise genome engineering. Many small molecule compounds can induce cell cycle arrest at different phases. Regarding HDR-preferred G2/M phase, at least two categories of compounds have cell cycle arrest effect at such phase. One is microtubule-active drugs, which can interrupt cell mitosis through inhibiting or stabilizing formation of microtubules, the key cellular component carrying out chromosomal segregation in mitosis. Microtubule-active drugs usually block cell cycle at G2 to M boundary [7, 8]. The other is DNA-damaging agents, typically including topoisomerase I inhibitors and alkylating agents. The DNA-damaging agents interrupt topoisomerase I action or produce interstrand DNA cross-links to inhibit DNA replication, thereby resulting in cell cycle arrest at G2/M phase [9, 10]. As a typical HDR enhancer, NOC is a microtubule inhibitor that has been widely investigated and applied for its HDR-enhancing effect [6, 11, 12]. However, many other small molecule compounds belonging to the above-mentioned categories are not studied for their activity on DNA repair and genome editing. Particularly, whether both the two types of cell cycle inhibitors can increase HDR remains unclear, and how they modulate DNA repair through certain molecular mechanisms needs further study.

Here we report the HDR-modulating effect of multiple cell cycle inhibitors, irinotecan (IRI), an analogue of classical topoisomerase I inhibitor camptothecin [9], mitomycin C (MITO), an alkylating agent [13], Docetaxel (DOC), a microtubule stabilizer [14], and NOC. The first two (IRI and MITO) are DNA-damaging agents and the latter two (DOC and NOC) are microtubule-active drugs. We investigate the cellular DNA repair response upon their treatment using various HDR reporter systems and animal cell lines. We also detect the cell changes in transcriptional profile upon small molecule treatment, with the aim to investigate the possible molecular targets and underlying mechanism of the small molecule effects.

## Results

### Effects of cell cycle inhibitor on CRISPR/Cas9-mediated KI

**Fig. 1:**
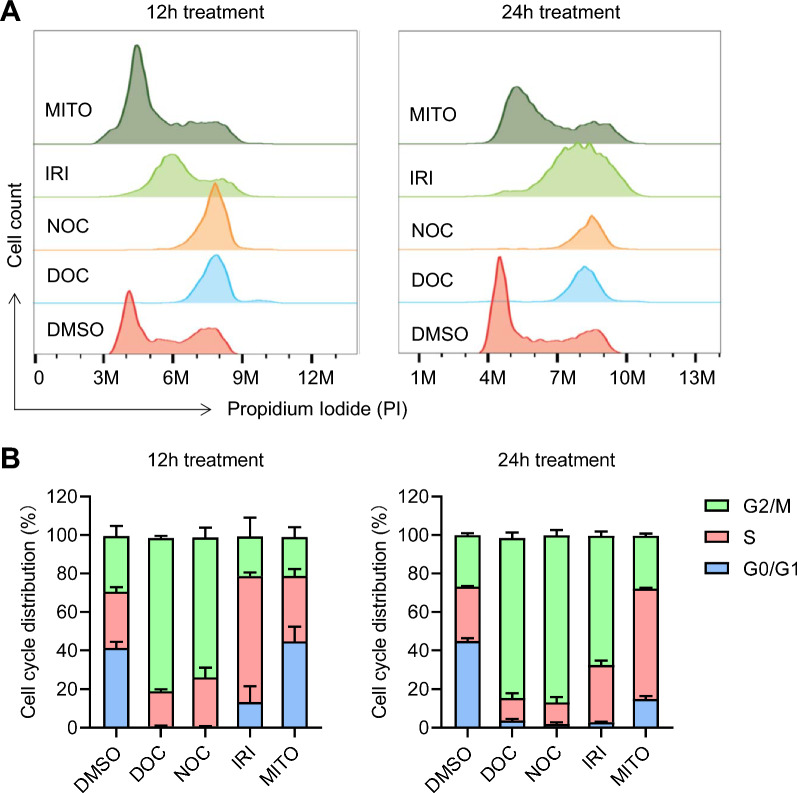
Cell cycle distribution analysis of 293T cells treated with small molecule inhibitors. **A** Analysis of 293T cells unsynchronized (DMSO) and synchronized by DOC (5 μM), NOC (2.5 μM), IRI (10 μM) and MITO (5 μM) for 12 (left panel) and 24 h (right panel) showing DOC and NOC arrested cells at G2/M stage and IRI and MITO increased the proportions at both S and G2/M phases of cell cycle. **B** Quantification of cell cycle distribution by measuring the area representing the specific cell cycle stage in FACS histogram. The mean values and error bars (SD) were calculated from three experiments

We first investigated the activity of the used small molecules on cell cycle synchronization. The 293T cells were treated with the 4 small molecules (5 μM DOC, 2.5 μM NOC, 10 μM IRI and 5 μM MITO) separately, and cell cycle distributions were analyzed by flow cytometry. We found DOC and NOC had great cell cycle synchronization activity with the most cells were arrested at G2/M phase after 12 h treatment. IRI and MITO also significantly increased the proportion of cell distribution at G2/M and S phases after 24 h treatment (Fig. 1A and B).

The effects of cell cycle inhibitor on CRISPR/Cas9-mediated KI was tested in the plasmid-based HDR reporter systems, in which the donors harboring the EGFP reporter and homology arms were used to insert EGFP into the 3’ end of GAPDH gene locus for a fused expression or repair the mutated EGFP by HDR. The donors were used in either double-stranded DNA (dsDNA, circular or linear) or single-stranded oligonucleotide (ssODN) form (Additional file 3: Fig. S1). After transfection and chemical treatment, flow cytometry was used to detect and quantify the fluorescence intensity, which represents the efficiency for KI-mediated EGFP expression.

**Fig. 2:**
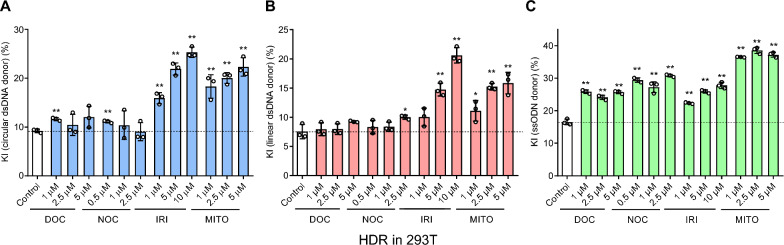
HDR-promoting effect of the four small molecules in 293T cells. The 293T cells were transfected with CRISPR/Cas9 and circular dsDNA (**A**), linear dsDNA (**B**) and ssODN donor (**C**)-mediated KI reporter system as shown in Additional file 3: Fig S1. After 12 h-transfection, the four small molecules were used to treat 293T cells for 48 h. The KI efficiency is shown by the percentage of EGFP-positive cells determined by flow cytometry. Data are mean ± SD. Each dot represents an independent experiment. **P < 0·01 and *P < 0·05 vs DMSO-treated control

By using the EGFP reporter system, we found that IRI ranged from 1 to 10 μM increased both dsDNA (circular or linear) and ssODN-mediated KI efficiency in a dose-dependent manner in 293T and BHK-21 cells (Fig. 2 and Additional file 3: Fig. S2A). DOC (1–5 μM), NOC (0.5–2.5 μM), and MITO (1–5 μM) increased KI with varying degree in the tested dose ranges. We also tested their activity in primary cultured cells, pig fetal fibroblasts (PFFs). As primary cells were more vulnerable to the small molecules, we used a lower dose range and found a dose-dependent KI-promoting effect for all the small molecules (Additional file 3: Fig. S2B).

We next tested whether combinations of these small molecules can further enhance KI. In general, combinational use of the small molecules generated enhanced KI effect than individual use of them, and the highest KI could be achieved in combinational use of 3 or 4 small molecules in most tested cells with various donors (Additional file 3: Fig. S3). Interestingly, a cell type-specific effect of the small molecules could be found. For example, in the same experimental conditions, IRI and MITO were more active than DOC and NOC in 293T cells to increase HDR (Fig. 2). In contrast, DOC and NOC were more active than IRI and MITO in BHK-21 and PFFs (Additional file 3: Fig. S2A and B). These results imply that the small molecule effect on promoting KI was profoundly influenced by cell types.

### Production of KI cells and embryos by cell cycle synchronization

**Fig. 3:**
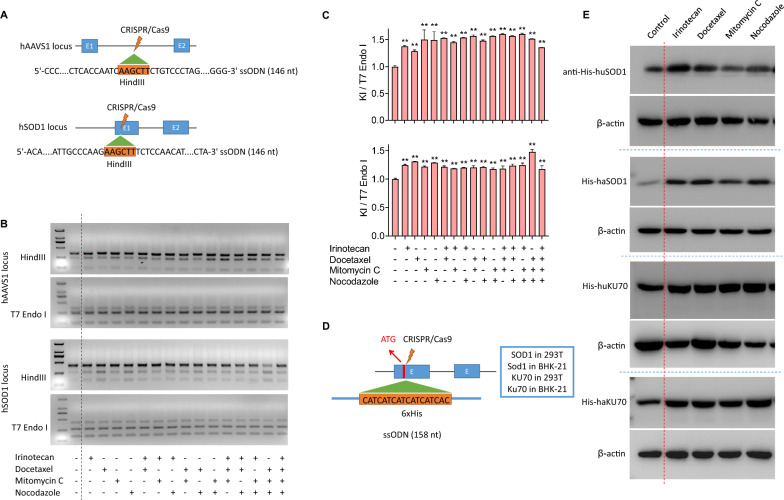
Small molecule effects on ssODN-mediated KI in endogenous genes of cells. **A** The donor is a 146 nt ssODN that is homologous to the target sequence and contains a 6 nt insertion (HindIII restriction sequence) at the CRISPR cleavage site for a simple identification of positive KI alleles by HindIII digestion. **B** The KI frequency after 48 h-treatment with different small molecules was determined directly by HindIII digestion of PCR products covering the KI site. The ratio of cleaved products to total DNA substrate (cleaved PCR bands + uncleaved PCR band) is KI frequency, and a T7E1 digestion of the same PCR product was used an inner control to show all mutant alleles (NHEJ + HDR). **C** Quantification of KI frequency (up, AAVS1 locus; down, SOD1 locus) in 293T cells with different small molecule treatments by estimating band density shown in **B** by Image J software. The mean values and error bars (SD) were calculated from three experiments. **P < 0.01 compared to control group. **D** The strategy for gene tagging by CRISPR-induced ssODN-mediated KI. A 158 nt ssODN donor for tagging a 6 × His epitope in the N terminal of two genes (SOD1 and KU70) in 293T and BHK-21 cells. **E** The ssODN-mediated protein tagging effects were determined by western blot. Representative results showing increased 6 × His tagged proteins in small molecule-treated groups compared to DMSO-treated control

We next tested the activity of cell cycle inhibitors on increasing KI in various endogenous gene loci. We assayed four 146 nt ssODN templates spanning AAVS1, SOD1, Apoe and Sox2 target sequences of 293T and BHK-21 cells (Fig. 3A and Additional file 3: Fig. S4A). ssODN-mediated KI efficiency was detected by HindIII digestion of the PCR products covering the target regions. Besides, T7E1 digestion of the same PCR products could represent all targeting events including HDR and NHEJ. All tested small molecules and their mix treatment demonstrated markedly increased HindIII-digested bands compared to control, suggesting increased proportion of ssODN-mediated KI cells in the treated cells (Fig. 3B and Additional file 3: Fig. S4B). Through quantifying the band density and comparing the KI rates (represented by HindIII digestion rate/T7E1 digestion rate), 1.2–1.5-fold enhancement in HDR could be observed after small molecule treatment (Fig. 3C and Additional file 3: Fig. S4C). We also used CRISPR/Cas9 and 158 nt ssODN to tag a 6 × His fragment at N terminal of multiple genes (Fig. 3D). Western blot of the tagged protein by anti-His antibody showed increased tagging rate in small molecule-treated cells compared to control in all tested targets (Fig. 3E). We further confirmed the increased tagged protein abundance with small molecule treatment by immunofluorescence staining assay (Additional file 3: Fig. S5A and B).

Considering the practical use of the compounds for creating genetically modified animals, we treated animal embryos with small molecules to investigate whether they could enhance KI efficiency in embryos. As a research model, we used pig parthenogenetically activated embryos for CRISPR/ssODN donor injection. The Cas9 protein, gRNA and ssODN targeting pig Rosa26 locus were used to introduce a HindIII restriction site, and injected embryos were individually identified by PCR and HindIII digestion for the presence of KI alleles. Our results showed that the four small molecules significantly increased the KI frequency in pig embryos, producing nearly twofold increase in treatment with 5 µM IRI, 0.5 µM DOC or 0.5 µM MITO, and threefold increase with 0.1 µM NOC. Of note, 5 µM IRI and 0.1 µM NOC did not impair embryo development, but 0.5 µM DOC and 0.5 µM MITO severely reduced the blastocyst rate, indicating more pronounced embryo toxicity of the two compounds. Moreover, combinational use of IRI + MITO or DOC + NOC displayed more severe toxicity to embryos while not further increasing KI frequency significantly than either of them alone (Table 1).

**Table 1:** KI efficiency in pig parthenogenetic embryos treated with small molecules

Treatment	Injected embryos	2-cell (%)	Blastocysts (%)	KI rate (%)
Control	466	77.52 ± 3.06^ab^	48.87 ± 4.84^c^	11.81 ± 1.84^a^
Irinotecan (5 µM)	460	81.12 ± 2.46^b^	46.97 ± 4.62^c^	23.61 ± 2.50^b^
Docetaxel (0.5 µM)	460	62.97 ± 3.36^ab^	19.14 ± 3.46^b^	23.61 ± 3.67^b^
Mitomycin C (0.5 µM)	447	70.81 ± 4.98^ab^	18.31 ± 4.66^b^	22.22 ± 2.78^b^
Nocodazole (0.1 µM)	302	75.53 ± 3.48^ab^	42.00 ± 4.59^c^	30.56 ± 1.39^C^
Irinotecan + Mitomycin C	120	70.83 ± 5.83^ab^	5.00 ± 1.67^a^	25.00 ± 25.00^abc^
Docetaxel + Nocodazole	184	56.14 ± 8.07^a^	16.14 ± 2.66^b^	30.56 ± 2.78^C^

KI rates are ratios of blastocysts with HindIII-cut alleles to all blastocysts

^a,b,c^Different letters indicate statistically significant differences between groups (mean ± SEM, P < 0.05)

### Transcriptomic changes upon cell cycle synchronization

**Fig. 4:**
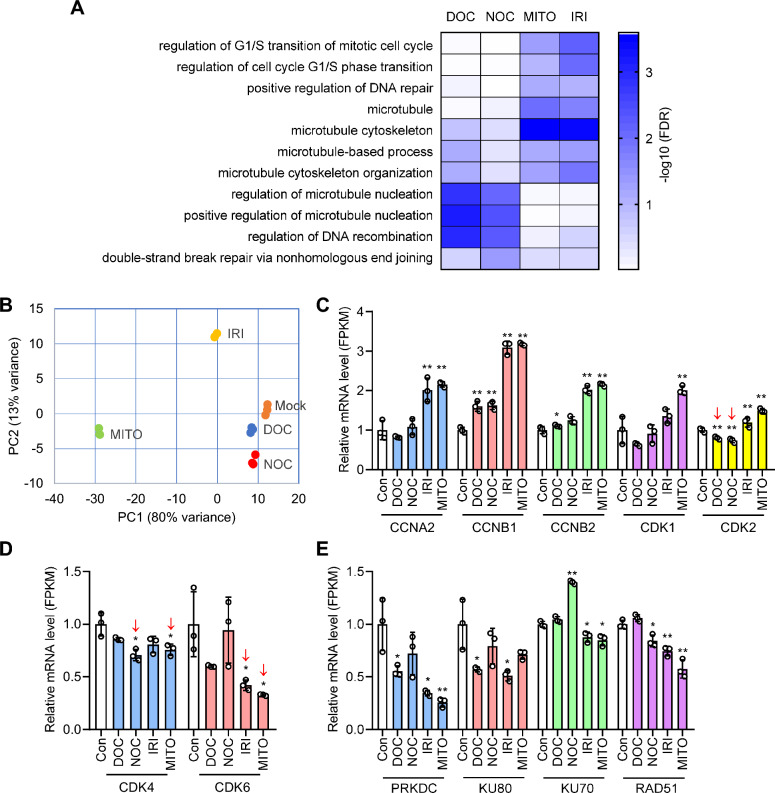
RNAseq analysis of 293T cells with cell cycle arrest with small molecules. **A** Gene Ontology (GO) analysis on RNAseq data showing significantly enriched GO terms in cell cycle, DNA repair and mitosis upon small molecule treatment. The differentially enriched GO terms implying the different action mechanism for cell cycle synchronization among the small molecules. **B** PCA showing IRI and MITO treatment groups clustered farther from the control than DOC and NOC groups, indicating the more profound change in transcriptome profile of cells with IRI and MITO treatment. **C** Cell cycle-associated genes, CCNA2, CCNB1/2, CDK1 and CDK2, are significantly upregulated in IRI and MITO treatment groups, whereas only CCNB1 had significant increase in mRNA level in DOC and NOC treatment groups. **D** CDK4 and CDK6 controlling G1 cell cycle phase showing insignificant difference or decrease in mRNA level of cells with small molecule treatment. **E** NHEJ factors (PRKDC, KU70 and KU80) and HDR factor (RAD51) showing decreased mRNA level in most groups with small molecule treatment. Gene expression levels were represented by normalized FPKM values. Data are mean ± SD from 3 independent samples. **P < 0·01 and *P < 0·05 vs DMSO-treated control

To investigate how the small molecules influence cell gene expression and related biological function, we performed RNAseq analysis in the cells treated with cell cycle inhibitors with DMSO treatment as control. The globe mRNA expression levels were shown in Additional file 1: Data S1. RNAseq analysis showed the small molecule treatment significantly affected the gene expression in cell cycle and DNA repair pathways, although at varying degrees among them (Fig. 4A). Principal component analysis (PCA) demonstrated that the global mRNA expression profiles of DOC- and NOC-treatment groups were close to the untreated control, whereas the IRI- and MITO-treatment groups formed distinct clustering far from the control, implying that IRI- or MITO-treatment provokes more pronounced changes in transcriptional profile in treated cells (Fig. 4B). We next specifically studied the expression level of genes controlling S and G2/M cell cycle phases. The mRNA levels of CCNA2 (cyclin A), CCNB1/2 (cyclin B), CDK1 and CDK2 showed significant increase in IRI and MITO treatment groups, but the increment in DOC and NOC treatment groups were less or insignificant (Fig. 4C). What’s more, the genes influencing other cell cycle phases, such as CDK4 and CDK6, had no significant change or reduced level in mRNA expression (Fig. 4D). As CCNA2/CDK2 and CCNB1/CDK1 are specifically enriched in late S and G2/M phases of cell cycles [15], the increase in their expression level implies a prolonged duration or cell cycle arrest in S and G2/M phase, creating a beneficial environment for HDR occurrence. The underlying molecular network for increased HDR in G2/M phase involves increased CCNB1/CDK1 expression or activity, which permits DNA end resection to favor HDR [16]. Furthermore, we found reduced mRNA expression of main NHEJ factors, KU80 and PRKDC, in most treatment groups, implying decreased NHEJ activity upon cell cycle synchronization (Fig. 4E).

### Cell cycle synchronization promotes activation of HDR factors

**Fig. 5:**
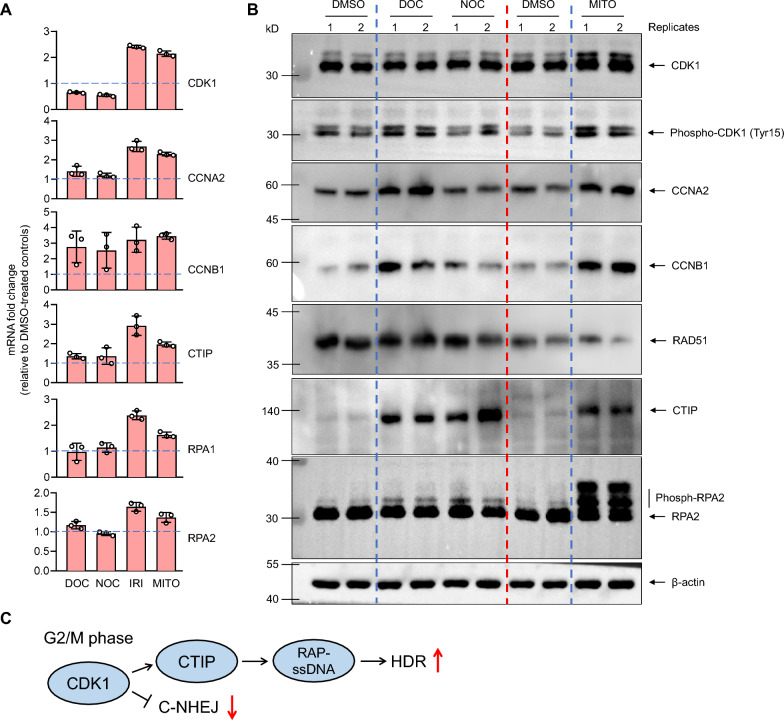
Analyzing the expression levels of genes in S-G2 cell cycle phases and HDR pathway by qPCR and WB. **A** qPCR assay showing the main factors controlling S and G2 progression (CDK1, CCNB1 and CCNA2) and HDR factors (CTIP, RPA1 and RPA2) exhibited greatly increased expression in 293T cells with IRI and MITO treatment compared to DMSO treatment control, whereas their up-regulation was less or insignificant in DOC and NOC treatment groups. Data are mean ± SD from 3 independent samples. **B** WB results showing similar gene expression changes as qPCR. CDK1 and RPA2 showing evident phosphorylation in all small molecule treated cells. **C** The data indicate cell cycle synchronization increases CDK1, CTIP and RPA2 activity to promote HDR. The small molecule inhibitors induce up-regulated CDK1 expression or activity, which promotes efficient DSB end resection by phosphorylating CITP and other nucleases and thus prevents DNA repair by NHEJ. Efficient break end resection generates sufficient ssDNA overhangs for RPA complex coating which is essential for HDR initiation

We used qPCR and WB to further investigate the gene expression alternations in cell cycle and DNA repair pathways. Results showed a similar expression level of CDK1, CCNB1 and CCNA2 as RNAseq data, with a more pronounced increase in cells treated with DNA-damaging agents (IRI and MITO) but less or no increase for microtubule-active drugs (DOC and NOC) (Fig. 5A and B, Additional file 3: Fig. S6, Additional file 2: Data S2). qPCR results found significantly increased mRNA level of HDR factors, CTIP, RPA1 and RPA2, in IRI and MITO treatment groups (Fig. 5A, Additional files 2, and 3: Data S2 and Fig. S6). Furthermore, WB results showed the increased protein abundance or phosphorylation of CDK1, CCNA2, CCNB1, CTIP and RPA2, although much less pronounced in DOC and NOC groups than that in IRI and MITO groups, indicating all the drugs can increase CDK1 activity to facilitate HDR in a similar molecular network but with different increasing extents (Fig. 5B), in agreement with the previously reported role of CDK1 as the key factor to bridge mitotic cell cycle and HDR process. The similar gene expression profiles indicate the common mechanism for HDR promoting effect with the cell cycle inhibitors. When cell cycle synchronization at G2 and S phases, accumulating CDK1 promotes phosphorylation of CtIP which is recruited to the DSB site for end resection, ssDNA generation and RPA binding. CDK1-dependent CTIP phosphorylation and activation controls the DNA repair switch from NHEJ to HDR in response to the small molecules that arrested cell cycle in G2 and S phases (Fig. 5C). Notably, the cell synchronization-mediated HDR promotion seems not RAD51 dependent as RAD51 had a generally decreased expression level upon the treatment (Figs. 4E and 5B).

### Overexpression of CCNA2, CCNB1 and CDK1 enhances KI frequency

**Fig. 6:**
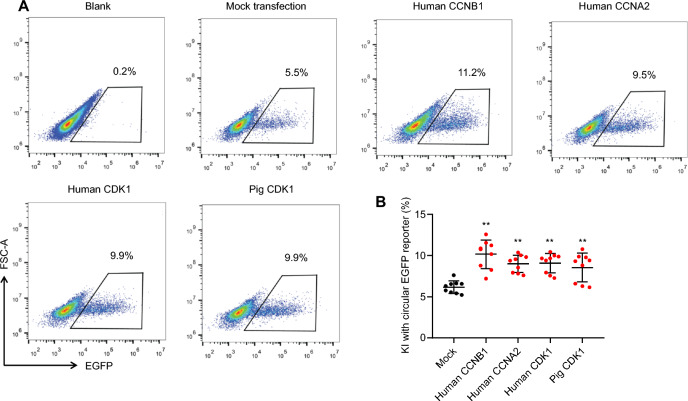
Overexpression of cell cycle factors increases KI frequency. **A** Overexpression of CCNA2, CCNB1 and CDK1 in 293T cells by transiently transfection of the eukaryotic expression vectors harboring cDNA of the genes. Enhanced KI frequency tested by further transfection of GAPDH-EGFP reporter shown in Additional file 3: Fig. S1 in overexpressing cells. **B** Overexpressing all tested genes in 293T cells showing promoting effect on KI, representing by increased proportion of EGFP-positive cells in CCNA2, CCNB1 and CDK1-transfected cells compared to mock transfection control. Data are mean ± SD of nine independent experiments. **P < 0·01 vs mock transfection control

To obtain direct evidence that enhanced protein abundance of the cell cycle-associated factors could promote HDR/KI efficiency, we successively transfected 293T cells with plasmids separately expressing CCNA2, CCNB1 and CDK1 (Fig. 6) and dsDNA EGFP KI reporter (Additional file 3: Fig. S1A). At 48 h after transfection, flow cytometry assay showed increased proportion of EGFP-positive cells which represents increased KI frequency in the overexpressing cells (Fig. 6A). Quantification of EGFP-positive KI cells showed that transfection of the G2/S-specific cell cycle factors promotes KI rate to 1.5–2 folds (Fig. 6B).

## Discussion

**Fig. 7:**
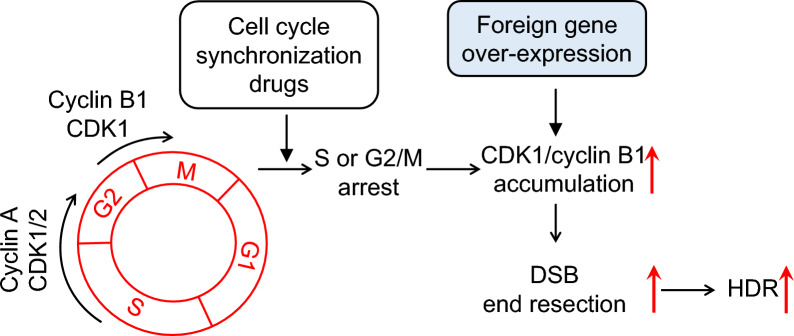
Model for the role of the small molecule inhibitors in enhancing HDR efficiency. The explanation is shown in main text

Our work proposed a model to elucidate the common effect of the cell cycle inhibitors on increasing HDR efficiency (Fig. 7). The small molecule inhibitors induce cell cycle arrest in S and G2/M stage, increasing protein abundance and activity of the cell cycle-specific factors, CCNB1/CDK1. In DSB repair process, CDK1 activates HDR factors to facilitate effective initiation of end resection, which is a critical determinant to activate HDR process. As the key genes communicating S-G2 cell cycle progression and HDR DNA repair, forced expression of CDK1, CCNB1 and CCNA2 by gene transfection can also achieve significant improvement of HDR efficiency.

DNA repair events are influenced by cell cycle status. It is generally considered S and G2/M phases of cell cycle is favorable for HDR occurrence. Many previous works found the cross-talk between the HDR and cell cycle machinery at S-G2/M phases [16–20]. CDK1 is the key component activated and accumulated in late S and G2/M phases to govern cell cycle progression in this stage. CDK1 activation is required for cell entry into S phase and mitosis. Likewise, CDK1 activity is also required for HDR upon formation of DSB in genome. In this aspect, CDK1 phosphorylates CTIP to initiate an efficient DSB end resection, promoting generation of ssDNA that is needed for HDR occurrence [17, 19, 21]. Besides, CDK1 can mediate phosphorylation of many proteins involved in DNA repair process, including Rad53 and Rad9 in fission yeast [22, 23] and BRCA2 in human cells [24]. Therefore, modulation of cell cycle affects DNA repair output. Cell cycle synchronization or arrest at G2/M phase refers to a persist activation of CDK1 protein, which favors initiation of HDR. As HDR is a precise but inefficient DNA repair form for many applications, regulation of cell cycle process provides a simple strategy to control DNA repair pathway to generate desired gene editing result.

To this end, we studied multiple small molecule cell cycle inhibitors with respect to their effect on CRISPR-mediated HDR in animal cells and embryos. The major two categories of compounds regulating mitotic cell cycle are microtubule-active drugs and DNA-damaging agents. They two affect DNA replication or cell mitosis by interacting with different cellular targets. Microtubule-active drugs target microtubule to prevent mitosis, resulting in cell arrest at the G2/M, whereas DNA-damaging agents target various DNA replication machinery to interrupt DNA synthesis. Although DNA-damaging agents exert their effect mainly in S phase, they commonly induce G2/M arrest through reciprocal action between cell cycle and DNA repair, including DNA damage increase, replication fork stalling and CCNB1/CDK1 accumulation [10]. Although mechanisms differ between the two types of drugs, common key cellular factors respond to them to control cell cycle running. CCNB1/CDK1 complex regulates G2/M transition. CCNB1 accumulates starting from S phase to activate CDK1, progressing cell cycle into mitosis. After mitosis, CCNB1 is rapidly degraded to trigger mitotic exit. Upon cells treated with DNA-damaging agents, CCNB1 degradation rate is decreased, resulting in a continuing accumulation of CCNB1. Overexpressed CCNB1 delays mitotic exit and prolonged mitotic arrest [10, 25]. For microtubule inhibitors, CCNB1/CDK1 level or activity is also the determinant factor to govern cell fate in mitosis. Upon microtubule inhibitor treatment, CCNB1 is slowed degraded, thus cannot fall below the “threshold” required for mitotic exist in many cell populations [7, 8, 26]. From the common point, CCNB1/CDK1 are both overexpressed and activated beyond the normal G2/M period, resulting in G2/M arrest. As a result, continuing activated CDK1 increases resection of DSB ends, therefore increases HDR frequency. From WB results in Fig. 5B, phospho-CDK1 was increased for both microtubule-active drug (DOC and NOC) and DNA-damaging agents (MITO), clearly indicating activated CDK1 upon treatment. The increasing level of phospho-CDK1 was more pronounced in MITO than DOC/NOC, reflecting their different mechanisms in activating CCNB1/CDK1. Likewise, phospho-RPA2 which represents resected ssDNA levels also had similarly increased level as phospho-CDK1, directly reflecting HDR activation after drug treatments [27].

It is noteworthy that the two types of small molecules possess distinctive capabilities in promoting HDR. In 293T cells, MITO and IRI treatment induced more robust increase in HDR efficiency than DOC and NOC (Fig. 2), in agreement with their varying mRNA and protein levels in the factors of the CCNB1-CDK1-CITP-RPA axis in 293T cells (Fig. 5). We also observed that the small molecules showed a cell type-specific HDR promoting activity. Unlike 293T cells, MITO and IRI treatment was less potent than DOC and NOC to increase HDR in PFF cells (Additional file 3: Fig. S2B). The difference highlights a dedicate contrast in the way that the two types of small molecules regulate cell cycle progression and CCNB1/CDK1 protein accumulation levels, despite sharing a common signal pathway. Therefore, the small molecule effect should be analyzed to identify better HDR enhancers when used in untested cells or organisms.

In summary, we report that cell cycle arrest at G2/M phases by various small molecule compounds can promote HDR frequency in animal cells. The key factor centered in the cell cycle arrest-induced HDR is CDK1, which is overexpressed by different small molecule effects to prolong G2/M phase and initiate the occurrence of HDR pathway. Our study expands the small molecule pools that have HDR promoting activity. However, the safety of these small molecules should be substantially studied as respect to the cytotoxicity and genotoxicity to assess the potential use in genome engineering in cells and animals.

## Materials and methods

### Cell lines

293T and BHK-21 (ATCC) were grown in DMEM (high glucose, Thermo Fisher Scientific) supplemented with 10% Fetal Bovine Serum (Thermo Fisher Scientific) and GlutaMAX (Thermo Fisher Scientific) at 37 °C with 5% CO_2_. Primary PFFs were isolated from a 30-day pig fetus, and grown in DMEM (high glucose) supplemented with 15% Fetal Bovine Serum and GlutaMAX at 39 °C with 5% CO_2_.

### Vectors and reporter system

Three types of homologous templates were constructed separately to perform circular dsDNA, linear dsDNA and ssODN-mediated KI by coupling with corresponding CRISPR/Cas9 vector (based on eSpCas9(1.1), Addgene plasmid #71814) in cell transfection. For dsDNA homologous templates, the plasmids containing EGFP flanked by approximately 800-bp homology arms on both left and right sides of GAPDH target were used to insert EGFP to C terminal of GAPDH for an in-frame expression. The ssODN-mediated KI system contains a mutated EGFP expression vector disrrupted by an inserted gRNA sequence and a stop codon in the middle of the EGFP gene, and an ssODN with phosphorthioate (PS) modification in both ends to restore an intact EGFP sequence [28, 29]. The following ssODNs were used for KI manipulation in endogenous genes: 146 nt PS-modified ssODNs for HindIII KI in AAVS1 and SOD1 loci in 293T cells, Apoe and Sox2 loci in BHK-21 cells and ROSA26 locus in pig embryos; 158 nt PS-modified ssODNs for 6 × His tagging in N terminal of SOD1 and KU70 in 293T and BHK-21 cells. CDS sequences of human CCNA2, CCNB1, CDK1 and pig CDK1 were amplified by RT-PCR from cDNA of corresponding cells and cloned into pcDNA3.1(+) vector (Invitrogen) for overexpression. The ssODN, CRISPR-gRNA and related primer sequences were listed in Additional file 3: Table S1.

### KI efficiency assay

The dose-dependent effect and additive effect of small molecules were tested in multiple immortal cell lines including 293T and BHK-21 and primary cells from pig embryos. Cells were subcultured in suitable wells one day prior to transfection and transfected with donor and CRISPR/Cas9 vectors targeting corresponding loci with Lipofectamine 3000 (Thermo Fisher Scientific) according to the manufacturer’s manual. Small molecules (Nocodazole, HY-13520; Docetaxel, HY-B0011; Irinotecan, HY-16562; and Mitomycin C, HY-13316. All from MCE) were added into cell culture at 12 h after transfection and cells were further incubated for 2 days to assay KI efficiency. The KI efficiency was represented by EGFP-positive rate determined by flow cytometry. The small molecule concentrations used in cells are 10 µM for irinotecan, 5 µM for docetaxel, 5 µM for mitomycin C, 2.5 µM for nocodazole and their mix except for the dose gradients indicated in Fig. 2 and Additional file 3: Fig. S2.

### Screening of ssODN-mediated KI cells

Cells were transfected with ssODN and CRISPR shown in “Vectors and reporter system” section and treated with small molecules for 48 h to identify KI efficiency. For HindIII KI in AAVS1 and SOD1 loci in 293T cells, as well as Apoe and Sox2 loci in BHK-21 cells, the primers were designed to amplify DNAs covering the HindIII restriction site introduced into the modified alleles (HindIII KI site PCR primers**,** Additional file 3: Table S1). KI efficiency was identified by HindIII digestion of PCR products, using T7E1 digestion as a control showing all editing events. For T7E1 assay, PCR product were hybridized in NEB Buffer 2 (New England Biolabs) using the following conditions: 95 °C for 5 min, 95–85 °C at − 2 °C/s, 85–25 °C at − 0.1 °C/s and held at 4 °C. Then, the hybridized PCR products were cut by T7E1 enzyme (New England Biolabs) and gel analyzed to determine the percent of total editing rate in the target alleles. The KI rate was calculated with HindIII digestion rate/T7E1 digestion rate. For detecting tagging efficiency of 6 × His at N terminal of SOD1 and KU70 in 293T and BHK-21 cells, cells were lysed for western blot or fixed for immunofluorescence using anti-His antibody after small molecule addition to the transfected cells for 48 h.

### Embryo injection

Pig embryo collection, culture and injection process were conducted as previously described [30]. In brief, pig ovaries were collected from a local slaughter house and cumulus oocyte complexes (COCs) were aspirated from antral follicles. After 3 times washing in maturation medium, COCs were cultured in a 4-well multi-dish (Nunc) at 39 °C in maturation medium in an atmosphere of 5% CO_2_ in air for 42 to 44 h. After the maturation culture, oocytes were freed from cumulus cells by vigorous vortexing. Cumulus-free oocytes were activated by an electric pulse, followed by 4 h of incubation in embryo culture medium (PZM3) containing 2 mM 6-Dimethylaminopurine (Sigma). The activated parthenogenetic embryos were subjected to cytoplasmic microinjection at 1-cell stage with the mixture containing spCas9 nuclease protein (New England Biolabs), synthesized Rosa26 sgRNA and ssODN donor at 300, 50 and 25 ng/μl, respectively. Approximately 10 picoliters of mixture was injected into each embryo. Injected embryos were washed three times in PZM3 and transferred to a 4-well multi-dish containing the same medium with small molecules and incubated at 39 °C in 5% CO_2_ in air for 6 days to reach blastocyst stage. Embryo was individually examined by PCR amplification of target site and HindIII digestion to determine modification status. All reagents for embryo manipulation are from Sigma unless otherwise stated.

### RNAseq

The four small molecule-treated 293T cells and DMSO-treated control cells were subjected to total RNA extraction and RNAseq. In brief, the polyA mRNA was isolated for next generation sequencing library preparation. The libraries were loaded on Illumina NovaSeq 6000 and sequencing was carried out using a 2 × 150 bp paired-end configuration. The sequencing data were processed and aligned to Homo Sapiens reference genome (GRCh37) for further analysis of gene expression level, PCA, differentially expressed genes and functional enrichment. The sequencing was processed and analyzed by Azenta Life Sciences.

### Quantitative PCR

Total RNA was harvested from cell with TRIzol (Thermo Fisher Scientific) and reverse transcribed with PrimeScript RT reagent Kit with gDNA Eraser (Takara). qPCR was performed using TB Green Premix Ex Taq (Takara) in a QuantStudio 5 Real-Time PCR System (Thermo Fisher Scientific) with the gene-specific primers (Additional file 3: Table S1). The relative mRNA expression of the tested genes was calculated using the 2^(−ΔΔCt)^ method by normalizing to the ACTB mRNA level.

### Western blot

Cell samples were homogenized with RIPA buffer (Thermo Fisher Scientific) supplemented with protease and phosphatase inhibitor cocktail (Sigma). Cell lysate supernatants were quantified with Pierce BCA Protein Assay Kit (Thermo Fisher Scientific) and adjusted to an identical concentration using H_2_O. Equal amount of samples was boiled in SDS-PAGE loading buffer and loaded for SDS-PAGE. After protein separation in gel, proteins were transferred on a PVDF membrane (Millipore) under wet condition. The membrane was blocked in 5% non-fat milk in TBST buffer and then incubated with primary antibody labeling specific protein. After washing three times with TBST, the membrane was further incubated with HRP conjugated secondary antibody specific to the IgG of the species of primary antibody host. The target proteins were visualized with ECL (Thermo Fisher Scientific). The primary antibodies used in this study are as follows: anti-His-Tag (66005-1-Ig, Proteintech), anti-CCNA2 (18202-1-AP, Proteintech), anti-CCNB1 (55004-1-AP, Proteintech), anti-CDK1 (19532-1-AP, Proteintech), anti-Phospho-CDK1 (Tyr15) (#4539, Cell Signaling), anti-RAD51 (14961–1-AP, Proteintech), anti-CTIP (12624-1-AP, Proteintech), anti-RPA2 (10412-1-AP, Proteintech) and anti-β-actin (sc-47778, Santa Cruz Biotechnology).

### Immunofluorescence

BHK-21 cells were transfected with ssODN containing 6 × His tag and CRISPR/Cas9, and treated with the small molecules for 48 h. Cells were fixed with 4% paraformaldehyde for 15 min followed by washing three times in PBS for 5 min each time. Then, cells were blocked in immunostaining blocking solution for 60 min followed by incubating cells with His-tag antibody at appropriate dilution in PBS overnight at 4 °C. After rinsing three times in PBS for 5 min each time, cells were further stained with fluorescence-conjugated secondary antibody at appropriate dilution in PBS for 1 h at 37 °C in darkness. Finally, the cells were thoroughly washed with PBS and examined by fluorescence microscopy.

### Cell cycle

Cell cycle distributions were determined by flow cytometry. 293T cells with small molecule treatments were trypsinized to single cells, washed in PBS and fixed in 70% ethanol at 4 °C for 30 min. The cells were then washed twice in PBS and incubated in a solution containing 20 μg/ml propidium iodide (PI, Sigma), 100 μg/ml RNase A (Sigma) and 0.05% Triton X-100 (Sigma) for 40 min in darkness at room temperature. Cell fluorescence was measured using an Accuri C6 Flow Cytometer (BD Biosciences). The cell areas representing G0/G1, S and G2/M phases in a DNA histogram were labeled and calculated.

### Data analysis

All data are presented as mean values ± SD unless otherwise indicated. The means and SD are calculated from values of independent experiments, which are labeled as circles or dots on each bar in the plots unless otherwise indicated in figure legends. Statistical significance was determined by Student’s t test analysis (two-tailed) for two groups and one-way ANOVA with Dunnet’s post-hoc test for three or more groups. Differences in means were considered statistically significant at P < 0.05. Significance levels are: * P < 0.05; ** P < 0.01.

### Supplementary Information

**Additional file 1: Data S1**. RNAseq gene expression data normalized as Fragments Per Kilobase of transcript per Million mapped reads (FPKM).

**Additional file 2: Data S2**. qPCR RAW readout and data analysis.

**Additional file 3:** **Figure S1**. Reporter system monitoring HDR efficiency. **A** CRISPR/Cas9‐induced KI with dsDNA donor (circular or linear) with homology arms in GAPDH locus. LA, left arm, RA, right arm. **B** CRISPR/Cas9‐induced KI with ssODN donor to repair EGFP sequence. ssODN is chemically modified with PS linkage at both ends to optimize the DNA repair efficiency. The EGFP‐repaired ssODN sequence is shown in Table S1. **Figure S2**. Test of CRISPR/Cas9‐mediated HDR efficiency in BHK‐21 and PFF cells with DOC, NOC, IRI and MITO treatment. Circular dsDNA donor, linear dsDNA donor and ssODN donor were separately used in BHK‐21 (**A**) and PFF cells (**B**). The cells were transfected with CRISPR and reporter for 12 h and then treated with small molecules for 48 h. HDR efficiency is demonstrated by EGFP positivity tested by flow cytometry. Control are cells with reporter transfection and then DMSO treatment for the same time. Data are mean ± SD from 2 or 3 independent experiments. **Figure S3**. Test of the combinational use the four small molecules on HDR efficiency. The four small molecules in different combinations were used to treat 293T (**A**), BHK‐21 (**B**) and PFF (**C**) transfected with CRISPR/Cas9 and circular dsDNA, linear dsDNA or ssODN donor. HDR efficiency is shown by the percentage of EGFP‐positive cells. Control are cells with reporter transfection and then DMSO treatment for the same time. Data are mean ± SD. Each dot represents an independent experiment. **Figure S4**. Small molecule effects on ssODN‐mediated KI in Apoe and Sox2 loci of BHK‐21 cells. **A** The donor is a 146 nt ssODN that is homologous to the target sequence and contains a 6 nt insertion (HindIII restriction sequence) at the CRISPR cleavage site. **B** The KI frequency after 48 h‐treatment with different small molecules was determined by HindIII digestion of PCR products covering the KI site. The ratio of cleaved products to total DNA substrate (cleaved PCR bands + uncleaved PCR band) is KI frequency. A T7E1 digestion of the same PCR product was used an inner control to show all targeting events including HDR and NHEJ. **C** Quantification of KI frequency of cells with different small molecule treatments by estimating band density shown in **B** by Image J software. The mean values and error bars (SD) were calculated from three experiments. **P < 0.01 compared to DMSO‐treated control group. **Figure S5**. Immunofluorescence assay of protein tagging frequency with small molecule treatment. The strategy inserting 6 × His tag into N terminals of Sod1 (**A**) and Ku70 (**B**) genes in BHK‐21 cell. After 12 h‐transfection and then small molecule treatment for 48 h, cells were immunostained with anti‐His antibody to show the abundance of tagged proteins. Enhanced fluorescence signals [red for His‐SOD1 (**A**) and green for His‐KU70 (**B**)] can be found in small molecule‐treated cells compared to DMSO‐treated cells, demonstrating enhanced tagging frequency in the two loci by small molecule treatment. Scale bars: 50 μm. **Figure S6**. Raw data for qPCR test of mRNA expression shown in Fig. 5A. Data are mean ± SD from 3 or 4 technical replicates. **P < 0.01 compared to DMSO‐treated control. **Table S1**. Oligoes and primers used in this study.

## Data Availability

The RNAseq data have been deposited in the NCBI Sequence Reads Archive (SRA) database under BioProject PRJNA998575.
