# Supplementary material for: Modulation of cell cycle increases CRISPR-mediated homology-directed DNA repair
Source: Cell Biosci. 2023 Nov 25;13:215. doi: 10.1186/s13578-023-01159-4 (PMC10676593; doi:10.1186/s13578-023-01159-4)
Supplement: Supplementary file 3 — Additional file 3: Figure S1. Reporter system monitoring HDR efficiency. A CRISPR/Cas9‐induced KI with dsDNA donor (circular or linear) with homology arms in GAPDH locus. LA, left arm, RA, right arm. B CRISPR/Cas9‐induced KI with ssODN donor to repair EGFP sequence. ssODN is chemically modified with PS linkage at both ends to optimize the DNA repair efficiency. The EGFP‐repaired ssODN sequence is shown in Table S1. Figure S2. Test of CRISPR/Cas9‐mediated HDR efficiency in BHK‐21 and PFF cells with DOC, NOC, IRI and MITO treatment. Circular dsDNA donor, linear dsDNA donor and ssODN donor were separately used in BHK‐21 (A) and PFF cells (B). The cells were transfected with CRISPR and reporter for 12 h and then treated with small molecules for 48 h. HDR efficiency is demonstrated by EGFP positivity tested by flow cytometry. Control are cells with reporter transfection and then DMSO treatment for the same time. Data are mean ± SD from 2 or 3 independent experiments. Figure S3. Test of the combinational use the four small molecules on HDR efficiency. The four small molecules in different combinations were used to treat 293T (A), BHK‐21 (B) and PFF (C) transfected with CRISPR/Cas9 and circular dsDNA, linear dsDNA or ssODN donor. HDR efficiency is shown by the percentage of EGFP‐positive cells. Control are cells with reporter transfection and then DMSO treatment for the same time. Data are mean ± SD. Each dot represents an independent experiment. Figure S4. Small molecule effects on ssODN‐mediated KI in Apoe and Sox2 loci of BHK‐21 cells. A The donor is a 146 nt ssODN that is homologous to the target sequence and contains a 6 nt insertion (HindIII restriction sequence) at the CRISPR cleavage site. B The KI frequency after 48 h‐treatment with different small molecules was determined by HindIII digestion of PCR products covering the KI site. The ratio of cleaved products to total DNA substrate (cleaved PCR bands + uncleaved PCR band) is KI frequency. A T7E1 digestion of the [file 13578_2023_1159_MOESM3_ESM.pdf]

Additional file 3

Captions for supplementary figures and table

Figure S1. Reporter system monitoring HDR efficiency. **A**. CRISPR/Cas9-induced KI with dsDNA donor (circular or linear) with homology arms in GAPDH locus. LA, left arm, RA, right arm. **B**. CRISPR/Cas9-induced KI with ssODN donor to repair EGFP sequence. ssODN is chemically modified with PS linkage at both ends to optimize the DNA repair efficiency. The EGFP-repaired ssODN sequence is shown in **Table S1**.

Figure S2. Test of CRISPR/Cas9-mediated HDR efficiency in BHK-21 and PFF cells with DOC, NOC, IRI and MITO treatment. Circular dsDNA donor, linear dsDNA donor and ssODN donor were separately used in BHK-21 (**A**) and PFF cells (**B**). The cells were transfected with CRISPR and reporter for 12 h and then treated with small molecules for 48 h. HDR efficiency is demonstrated by EGFP positivity tested by flow cytometry. Control are cells with reporter transfection and then DMSO treatment for the same time. Data are mean ± SD from 2 or 3 independent experiments.

Figure S3. Test of the combinational use the four small molecules on HDR efficiency. The four small molecules in different combinations were used to treat 293T (**A**), BHK-21 (**B**) and PFF (**C**) transfected with CRISPR/Cas9 and circular dsDNA, linear dsDNA or ssODN donor. HDR efficiency is shown by the percentage of EGFP-positive cells. Control are cells with reporter transfection and then DMSO treatment for the same time. Data are mean ± SD. Each dot represents an independent experiment.

Figure S4. Small molecule effects on ssODN-mediated KI in Apoe and Sox2 loci of BHK-21 cells. **A**. The donor is a 146 nt ssODN that is homologous to the target sequence and contains a 6 nt insertion (HindIII restriction sequence) at the CRISPR cleavage site. **B**. The KI frequency after 48 h-treatment with different small molecules was determined by HindIII digestion of PCR products covering the KI site. The ratio of cleaved products to total DNA substrate (cleaved PCR bands + uncleaved PCR band) is KI frequency. A T7E1 digestion of the same PCR product was used an inner control to show all targeting events including HDR and NHEJ. **C**. Quantification of KI frequency of cells with different small molecule treatments by estimating band density shown in **B** by Image J software. The mean values

and error bars (SD) were calculated from three experiments. **P < 0.01 compared to DMSO-treated control group.

Figure S5. Immunofluorescence assay of protein tagging frequency with small molecule treatment. The strategy inserting 6 × His tag into N terminals of Sod1 (**A**) and Ku70 (**B**) genes in BHK-21 cell. After 12 h-transfection and then small molecule treatment for 48 h, cells were immunostained with anti-His antibody to show the abundance of tagged proteins. Enhanced fluorescence signals [red for His-SOD1 (**A**) and green for His-KU70 (**B**)] can be found in small molecule-treated cells compared to DMSO-treated cells, demonstrating enhanced tagging frequency in the two loci by small molecule treatment. Scale bars: 50 μm.

Figure S6. Raw data for qPCR test of mRNA expression shown in **Figure 5A.** Data are mean ± SD from 3 or 4 technical replicates. **P < 0.01 compared to DMSO-treated control.

Table S1. Oligoes and primers used in this study.

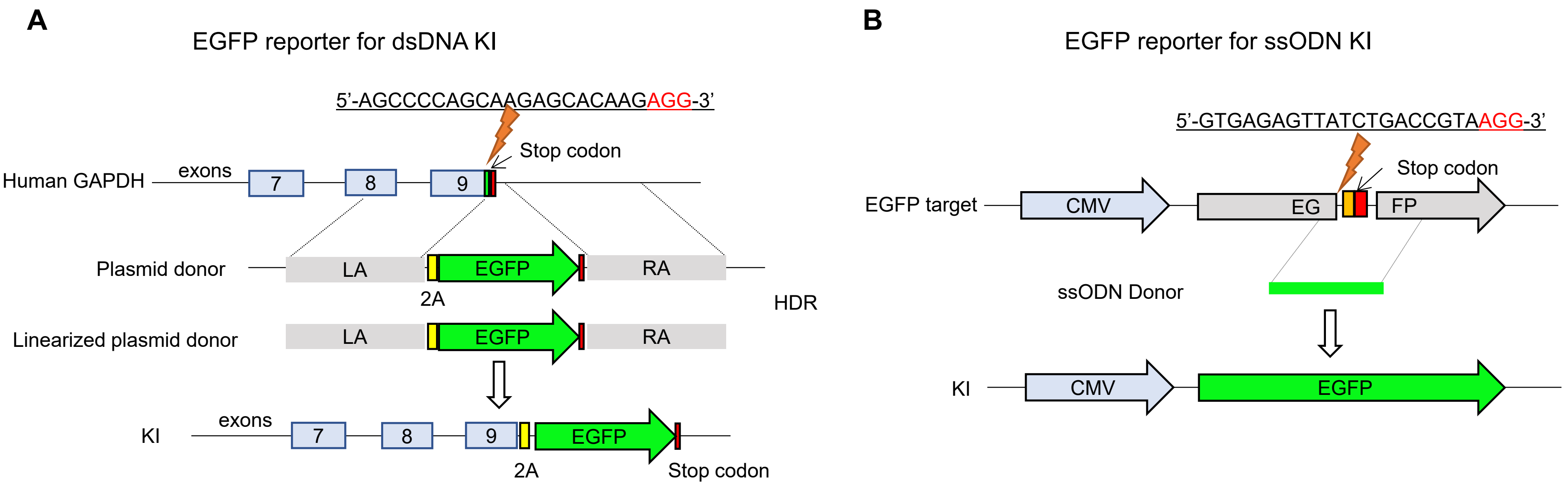
A
EGFP reporter for dsDNA KI
5'-AGCCCCAGCAAGAGCACAAGAGG-3'
Stop codon
Human GAPDH
exons
7
8
9
Plasmid donor
LA
EGFP
RA
2A
HDR
Linearized plasmid donor
LA
EGFP
RA
KI
exons
7
8
9
EGFP
2A
Stop codon
B
EGFP reporter for ssODN KI
5'-GTGAGAGTTATCTGACCGTAAGG-3'
Stop codon
EGFP target
CMV
EG
FP
ssODN Donor
KI
CMV
EGFP

Figure S1

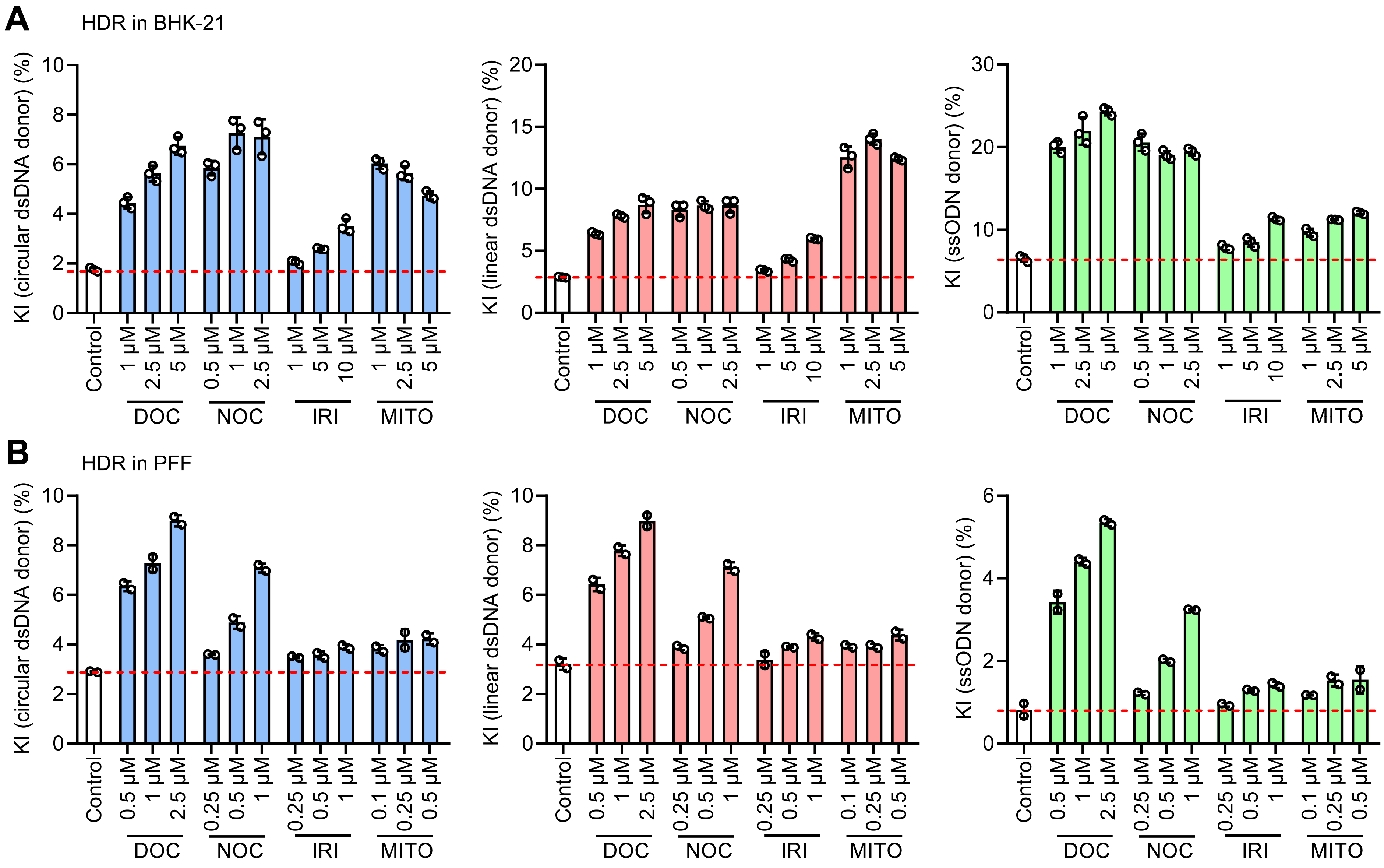
A
HDR in BHK-21
KI (circular dsDNA donor) (%)
KI (linear dsDNA donor) (%)
KI (ssODN donor) (%)
Control
1 μM
2.5 μM
5 μM
0.5 μM
10 μM
DOC
NOC
IRI
MITO
B
HDR in PFF
0.25 μM
0.1 μM

Figure S2

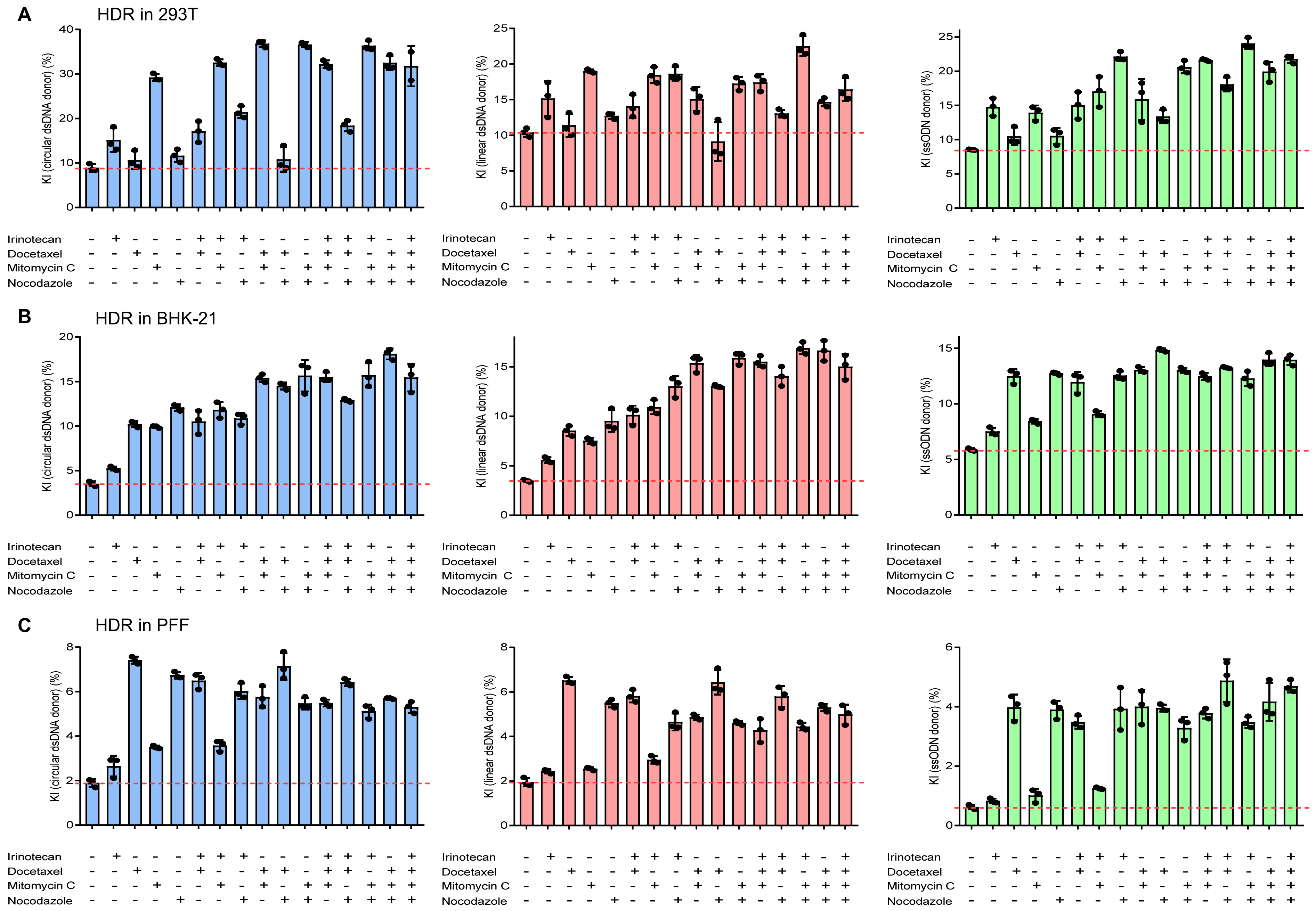

A
HDR in 293T
KI (circular dsDNA donor) (%)
KI (linear dsDNA donor) (%)
KI (ssODN donor) (%)
B
HDR in BHK-21
C
HDR in PFF
Irinotecan
Docetaxel
Mitomycin C
Nocodazole

Figure S3

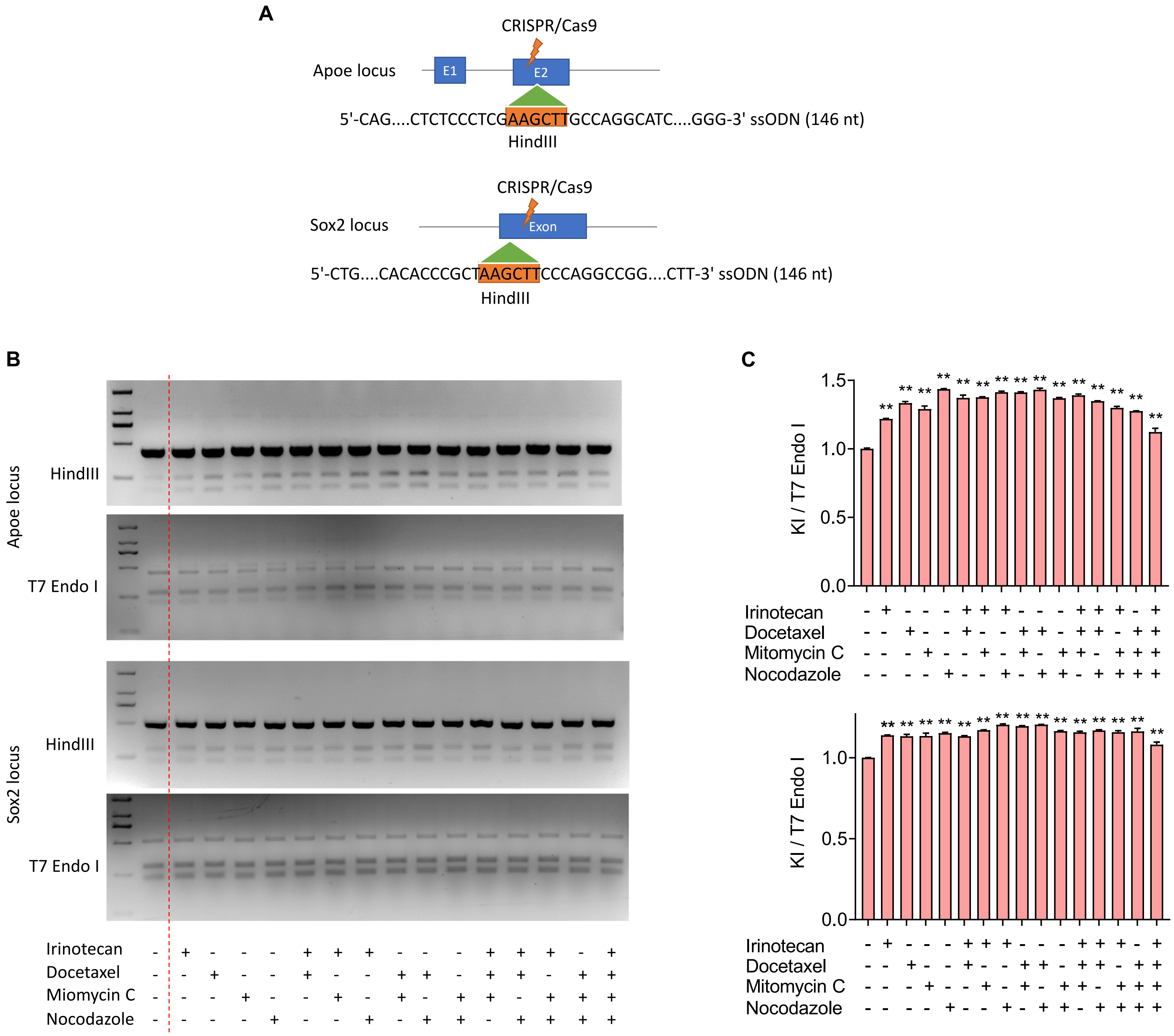

A
CRISPR/Cas9
Apoe locus
E1
E2
5'-CAG....CTCTCCCTCGAAGCTTGCCAGGCATC....GGG-3' ssODN (146 nt)
HindIII
CRISPR/Cas9
Sox2 locus
Exon
5'-CTG....CACACCCGCTAAGCTTCCCAGGCCGG....CTT-3' ssODN (146 nt)
HindIII
B
Apoe locus
HindIII
T7 Endo I
Sox2 locus
HindIII
T7 Endo I
Irinotecan
Docetaxel
Miomycin C
Nocodazole
C
KI / T7 Endo I
1.5
1.0
0.5
0.0
Irinotecan
Docetaxel
Mitomycin C
Nocodazole
KI / T7 Endo I
1.0
0.5
0.0
Irinotecan
Docetaxel
Mitomycin C
Nocodazole

Figure S4

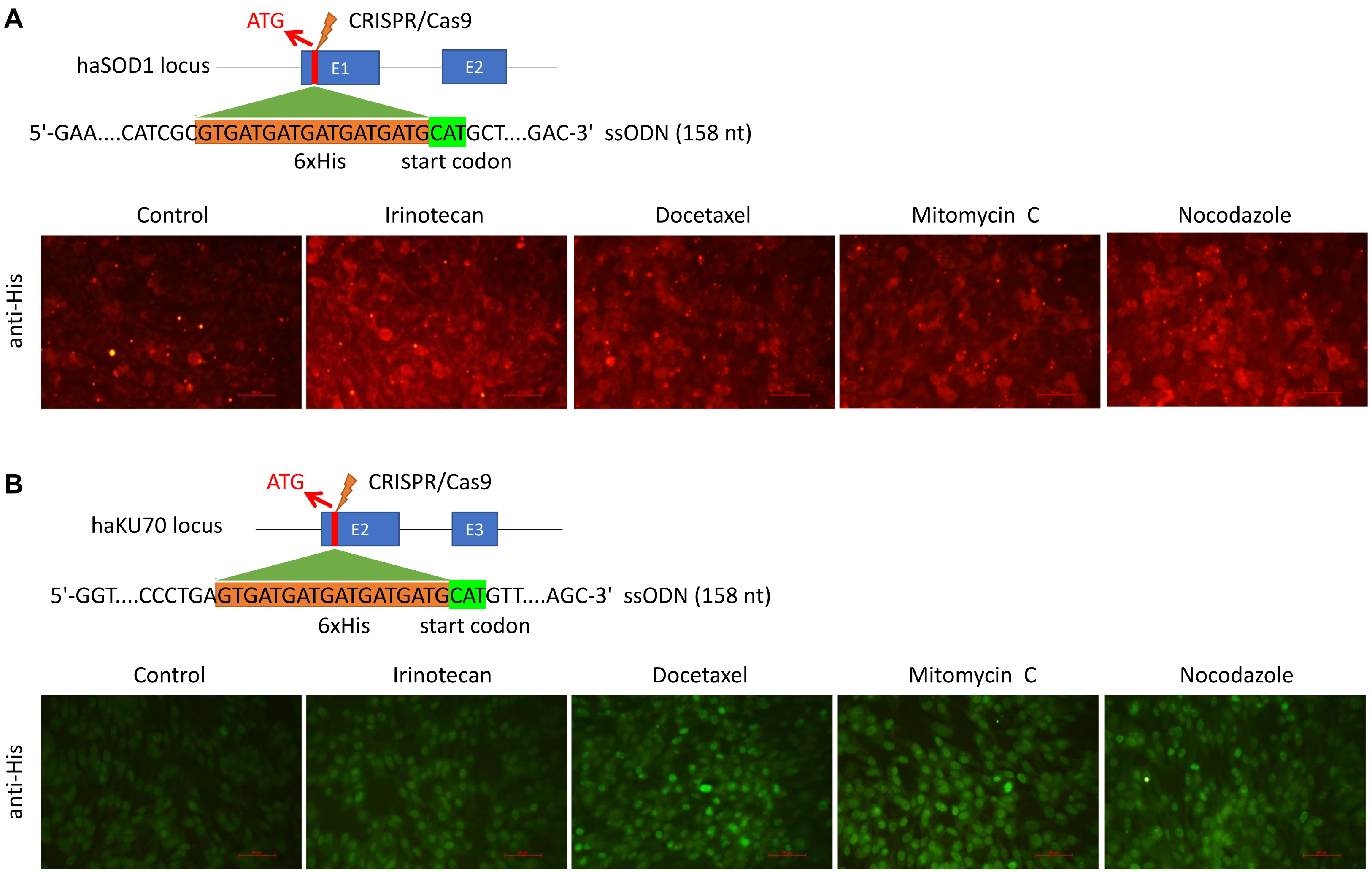
A
ATG
CRISPR/Cas9
haSOD1 locus
E1
E2
5'-GAA....CATCGCGTGATGATGATGATGATGCATGCT....GAC-3' ssODN (158 nt)
6xHis
start codon
Control
Irinotecan
Docetaxel
Mitomycin C
Nocodazole
anti-His
B
ATG
CRISPR/Cas9
haKU70 locus
E2
E3
5'-GGT....CCCTGAGTGATGATGATGATGATGCATGTT....AGC-3' ssODN (158 nt)
6xHis
start codon
Control
Irinotecan
Docetaxel
Mitomycin C
Nocodazole
anti-His

Figure S5

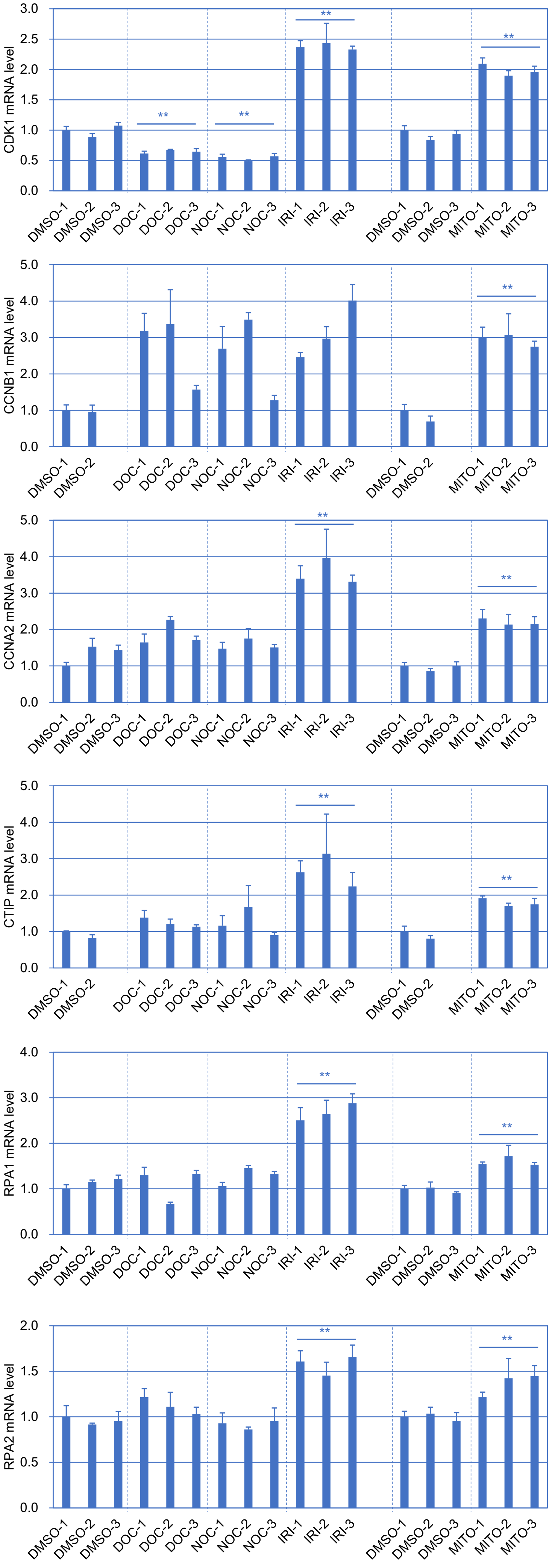
CDK1 mRNA level
CCNB1 mRNA level
CCNA2 mRNA level
CTIP mRNA level
RPA1 mRNA level
RPA2 mRNA level
**
DMSO-1
DMSO-2
DMSO-3
DOC-1
DOC-2
DOC-3
NOC-1
NOC-2
NOC-3
IRI-1
IRI-2
IRI-3
MITO-1
MITO-2
MITO-3

Figure S6

Table S1. Oligoes and primers used in this study

Application	Name	Sequence (5'-3')
KI in pig embryos and PFF	GAPDH gRNA	F: CACCGTCCAGGGGCTCTTACTCCT<br>R: AAACAGGAGTAAGAGCCCCTGGAC
	ROSA26 gRNA	F: CACCGTGAGAGTTATCTGACCGTA<br>R: AAACTACGGTCAGATAACTCTCAC
	ROSA26 ssODN	A-**PS**-T-**PS**-GTCTGGGACTGGATGAGCA<br>AGTACAACAAACAAAATGGGCTTAAAG<br>TATGAGTGAGAGTTATCTGACCAAGCT<br>TGTAAGGATGCAAGTGAGGGGGCCTA<br>AGGTTTGGAGATTAATATTTAATCTCAG<br>ATGCTATACTTTGGT-**PS**-G-**PS**-G
	ROSA26-HindIII KI site PCR primers	F: ATCGCCTCCATGTCAGTTTGCT<br>R: AAATGCACTGTTGGGCCTATGCTC
KI in 293T	GAPDH gRNA	F: CACCGAGCCCCAGCAAGAGCACAAG<br>R: AAACCTTGTGCTCTTGCTGGGGCTC
	6xHis-SOD1 gRNA	F: CACCGCGGCGTGGCCTAGCGAGTTA<br>R: AAACTAACTCGCTAGGCCACGCCGC
	6xHis-KU70 gRNA	F: CACCGAGCAGTAGCCAACATGTCA<br>R: AAACTGACATGTTGGCTACTGCTC
	AAVS1-HindIII gRNA	F: CACCGGGGCCACTAGGGACAGGAT<br>R: AAACATCCTGTCCCTAGTGGCCCC
	SOD1-HindIII gRNA	F: CACCGATTAGGCATGTTGGAGACTT<br>R: AAACAAGTCTCCAACATGCCTAATC
	6xHis-SOD1 ssODN	G-**PS**-A-**PS**-AATTGATGATGCCCTGCAC<br>TGGGCCGTCGCCCTTCAGCACGCACA<br>CGGCCTTCGTCGCGTGATGATGATGAT<br>GATGCATAACTCGCTAGGCCACGCCGA<br>GGTCCTGGTTCCGAGGACTGCAACGG<br>AAACCCCAGACGCTGCAGGAGACTAC<br>GAC-**PS**-G-**PS**-C
	AAVS1-HindIII ssODN	C-**PS**-C-**PS**-CAATATCAGGAGACTAGGA<br>AGGAGGAGGCCTAAGGATGGGGCTTT<br>TCTGTCACCAATCAAGCTTCTGTCCCTA<br>GTGGCCCCACTGTGGGGTGGAGGGGA<br>CAGATAAAAGTACCCAGAACCAGAGCC<br>ACATTAACCGGCCCTG-**PS**-G-**PS**-G
	SOD1-HindIII ssODN	A-**PS**-C-**PS**-AGAATCTTCAATAGACACAT<br>CGGCCACACCATCTTTGTCAGCAGTCA<br>CATTGCCCAAGAAGCTTTCTCCAACAT<br>GCCTAATAATGAAAAAGCATCAGATGG<br>ATTAGGGCTGATGCCACTAAACATCAA<br>GGTAGTTCATGAGC-**PS**-T-**PS**-A

	6xHis-KU70 ssODN	T-**PS-**T-**PS-**CTTGTTCTTCCTCTGCTTCT TCATCGCCCTCGGTTTTGTAATATGACT CCCACCCTGAGTGATGATGATGATGAT GCATGTTGGCTACTGCTCACTAGGCGA AAGACGTTAACGTCAGTAACAGGCAAA TTTAAATCGAAAAAATACCGACCTC**-PS-** T**-PS-**A
	SOD1-HindIII KI site PCR primers	F: AGAGCTGTATTTAGAATGCCTA<br>R: TCATTTTCACCGTAATTGTCC
	AAVS1-HindIII KI site PCR primers	F: CTCTCTAGTCTGTGCTAGCTC<br>R: ATAAGGAATCTGCCTAACAGGA
KI in BHK-21	Gapdh gRNA	F: CACCGAGGGTGGGTCTCTTACTCCT<br>R: AAACAGGAGTAAGAGACCCACCCTC
	6xHis-Sod1 gRNA	F: CACCGTCCCCTCGCGAAGCAAGCA<br>R: AAACTGCTTGCTTCGCGAGGGGAC
	Apoe-HindIII gRNA	F: CACCGCTCGAGCTCTCCCTCGGCC<br>R: AAACGGCCGAGGGAGAGCTCGAGC
	Sox2-HindIII gRNA	F: CACCGCTGGTTCACACCCGCTCCC<br>R: AAACGGGAGCGGGTGTGAACCAGC
	6xHis-Ku70 gRNA	F: CACCGCAAACCAACATGTCAGGGT<br>R: AAACACCCTGACATGTTGGTTTGC
	6xHis-Ku70 ssODN	G**-PS-**G**-PS-**TCTCCTCCTCTTCTTCCTCT TCCTCGCCCTCGGTTTTGTAGTAGGAT TCCCACCCTGAGTGATGATGATGATGA TGCATGTTGGTTTGCTCACTGGGTGAA CAACAAACAAATTTAAAAAGAAAAAACA CCGAGGAAAAGTACTTGTGTGCCAA**-P S-**G**-PS-**C
	6xHis-Sod1 ssODN	G**-PS-**A**-PS-**AGTGGATGGTGCCCTGCAC CGGGCCGTCGCCCTTCAGCACGCACA CGGCCTTCATCGCGTGATGATGATGAT GATGCATGCTTGCTTCGCGAGGGGAC GCGCGGCGGCCCCGGGAACCGGAGG ACGGCGAGGACACACCCGGCGACAAC GGAAG**-PS-**A**-PS-**C
	Apoe-HindIII ssODN	C**-PS-**A**-PS-**GGGTTTGCTGCTCTGCCAT GCCAGCTGCTCGTTTACCTCGGGCTCG AGCTCTCCCTCGAAGCTTGCCAGGCAT CCTGTGCAGAGTAAGTTCAAGGCTGGG TCAGGACCTACAATGCTCTTGGGTCTC TTTGGAGCGACAGTG**-PS-**G**-PS-**G
	Sox2-HindIII ssODN	C**-PS-**T**-PS-**GCCATTGCTCCAGCCGTTC ATGTGCGCGTAGCTGTCCATGCGCTG GTTCACACCCGCTAAGCTTCCCAGGCC

		GGCGCCTACCCCAACCCCGCTCGCCA TGCTGTTCCCGCCCGGGGCCAGCAGC CCTCCGGGAAGCGTGTAC**-PS-**T**-PS-**T
	Apoe-HindIII KI site PCR primers	F: AAGGTAGGTTTTTCTAATTCCATGC<br>R: AGTCGGAGACATTCCACCA
	Sox2-HindIII KI site PCR primers	F: TAAGATGGCCCAGGAGAACCC<br>R: CGAGCCGTTCATGTAGGTCT
ssODN-mediated EGFP reporter tested in all cells	EGFP gRNA	F: CACCGTGAGAGTTATCTGACCGTA<br>R: AAACTACGGTCAGATAACTCTCAC
	ssODN-PS modification	A**-PS-**C**-PS-**CCCGACCACATGAAGCAGC ACGACTTCTTCAAGTCCGCCATGCCCG AAGGCTACGTCCAGGAGCGCACCATCT TCTTCAAGGACGACGGCAACTACAAGA CCCGCGCCGAGGTGAAGTTCGAGGGC GACACCCTGG**-PS-**T**-PS-**G
qPCR primers for gene mRNA levels in 293T cells	CDK1	GAAGCCTAGCATCCCATGTC
		CCATTTTGCCAGAAATTCGT
	CCNB1	CACTTCCTTCGGAGAGCATC
		AGAAGGAGGAAAGTGCACCA
	CCNA2	CCTGCAAACTGCAAAGTTGA
		AAAGGCAGCTCCAGCAATAA
	CTIP	AGGTCAGACCATGGAGGATG
		AGGTCTGCTCCCGGATCTAT
	RPA1	TCAGGGTCAAAGTGGAGACC
		TGCTCCTCTCACATCAATGC
	RPA2	TTAAGATCATGCCCCTGGAG
		ATAGGTGCTCTCCCTGCTGA
	ACTB	GATGAGATTGGCATGGCTTT
		CACCTTCACCGTTCCAGTTT
